# Supplementary material for: Optimising knowledge mobilisation for mental health research in low- and middle-income countries: A systematic review of the state of knowledge and directions for future research
Source: Glob Ment Health (Camb). 2025 Sep 17;12:e106. doi: 10.1017/gmh.2025.10059 (PMC12509166; doi:10.1017/gmh.2025.10059)
Supplement: Faija et al. supplementary material [file S2054425125100599sup001.docx]

**Appendix 1.** A hierarchy flow diagram chart to justify reason for exclusion at full text screening

**Is this article a conference paper, thesis, dissertation, book chapter, opinion letter (i.e. non-peer reviewed article)?**

**Exclusion Criteria 1**

Assess article using Exclusion Criteria 2

Exclude article using Criteria 1

NO

YES

**Is this article a systematic review/protocol paper**

**(i.e., not a research paper)?**

**Exclusion Criteria 2**

Assess article using Exclusion Criteria 3

Exclude article using Criteria 2

NO

YES

**Exclusion Criteria 3**

**Has data from this article been conducted/data collected in a high-income country?**

Assess article using Exclusion Criteria 3

Exclude article using Criteria 3

NO

YES

**Is this paper topic outside mental health?**

Note: Papers to ‘prevent’ mental health problems focused on ‘wellbeing’ would be included. Papers focused on mental health for people with other conditions will also be included.

**Exclusion Criteria 4**

Assess article using Exclusion Criteria 5

Exclude article using Criteria 4

NO

YES

**Is this article focused on developmental disorders, autism, ADHD, dementia, substance abuse, conduct disorder, or paraphilia?**

**Exclusion Criteria 5**

Assess article using Exclusion Criteria 6

Exclude article using Criteria 5

NO

YES

**Is this article providing EVALUATION data on knowledge mobilization?**

Questions that will help to check criteria 6**:**

**6a) Does the paper include information about knowledge mobilization*?**

**YES** (Go to 6b) **NO** (Go to Criteria 7)

**6b) Can we extract information on barriers, facilitators, lessons learned to mobilise knowledge?**

**YES** (Include) **NO** (Exclude using Criteria 6

OR Go to Criteria 7)

**Exclusion Criteria 6**

***Knowledge Mobilization definition:**

"Knowledge mobilisation is activity that involves working with research users to make research usable and useful - the emphasis is on collaboration.

*We explicitly differentiate knowledge mobilisation from dissemination and from impact. Both these terms are commonly used by researchers and funders to talk about things that happen at the end of research – how the final work is disseminated (shared, published, publicised) and what effects this has. These are obviously important aspects of knowledge mobilisation – research cannot be useful if it is not shared, and ‘being useful’ is the impact we are aiming for. However, these can occur only at the end of the research cycle, and our definition recognises that working with users can happen at any point. Activities that happen at the beginning or during a research cycle, such as priority-setting with users, or co-designing interventions to be tested, can be considered knowledge mobilisation as they are about working with users to make decisions about what research would be useful, and how research can be carried out collaboratively with users'.*

Exclude article Exclusion Criteria 6

NO

YES

Assess article using Exclusion criteria 7 Criteria 6

NO

YES

**Other reason not listed in the criteria?**

**Exclusion Criteria 7**

Exclude article using Criteria 7 in COVIDENCE & Complete word table to note the reason for exclusion

Include the paper

**Appendix 2.** Search term strategy

| (1) Knowledge Mobilization Terms | | (2) Mental Health Terms | (3) LMIC Terms |
| --- | --- | --- | --- |
| (a) KM terms | (b) KM methods/  Strategies | General/CMHD or  Disorder specific | General/ Country specifics (OECD criteria) |
| "knowledge mobilization" or "translational research" or "knowledge translation" or "knowledge transfer" or "knowledge management" or "research utilisation" or "knowledge exchange" or "implementation" | "collaboration" or "personal relationships" or "outreach" or "audit" or "feedback" or "meeting" or "workshop" or "courses" or "conference" or "lecture" or "seminar" or "symposi*" or "education" or "intervention" or "dissemination" | "mental health" or "mental illness" or "mental disorder" or "psychiatric illness" or "anxiety" or "depress*" or "well-being" or "wellbeing" or "distress" or "emotional disorder" or "mood" or "stress" or "trauma" or "schizo*" or "psychotic" or "psychosis" or "dysthymia" or "affect" or "bipolar" or "phobia" or "panic" or "agora*" or "OCD" or "PTSD" or "personality" or "obsess*" or "compul*" | "developing countr*" or "developing nation" or "developing population" or "developing econom*" or "deprived countr*" or "deprived nation" or "deprived population" or "middle income countr*" or "middle income nation" or "middle income population" or "middle income level" or "middle income econom*" or "low* income countr*" or "low* income nation" or "low* income population" or "low* income level" or "low* income econom*" or "poor* countr*" or "poor* nation" or "poor* population" or "less* developed econom*" or "underdeveloped countr*" or "underdeveloped nation" or "underdeveloped population" or "underdeveloped econom*" or "under developed countr*" or "under developed nation" or "under developed population" or "under developed econom*" or "underserved countr*" or "underserved nation" or "underserved population" or "under served countr*" or "under served nation" or "under served population" or "third world countr*" or "low resource countr*" or "low and middle income countr*" or "low middle income countr*" or "low-and-middle income countr*" or "low-middle income countr*" or "LMIC" or "least developed countr*" or "Afghanistan" or "Albania" or "Algeria" or "Angola" or "Antigua and Barbuda" or "Argentina" or "Armenia" or "Azerbaijan" or "Bangladesh" or "Belarus" or "Belize" or "Benin" or "Bhutan" or "Bolivia" or "Bosnia and Herzegovina" or "Botswana" or "Brazil" or "Burkina Faso" or "Burundi" or "Cambodia" or "Cameroon" or "Cape Verde" or "Central African Republic" or "Chad" or "China" or "Colombia" or "Comoros" or "Congo" or "Costa Rica" or "Côte d’ivoire" or "Ivory Coast" or "Cuba" or "Djibouti" or "Dominica" or "Dominican Republic" or "Ecuador" or "Egypt" or "El Salvador" or "Eritrea" or "Eswatini" or "Swaziland" or "Ethiopia" or "Fiji" or "Gabon" or "Gambia" or "Georgia" or "Ghana" or "Grenada" or "Guatemala" or "Guinea" or "Guinea-Bissau" or "Guyana" or "Haiti" or "Honduras" or "India" or "Indonesia" or "Iran" or "Iraq" or "Jamaica" or "Jordan" or "Kazakhstan" or "Kenya" or "Kiribati" or "Korea" or "Kosovo" or "Kyrgyzstan" or "Lao" or "Lebanon" or "Lesotho" or "Liberia" or "Libya" or "Madagascar" or "Malawi" or "Malaysia" or "Maldives" or "Mali" or "Marshall Islands" or "Mauritania" or "Mauritius" or "Mexico" or "Micronesia" or "Moldova" or "Mongolia" or "Montenegro" or "Montserrat" or "Morocco" or "Mozambique" or "Myanmar" or "Namibia" or "Nauru" or "Nepal" or "Nicaragua" or "Niger" or "Nigeria" or "Niue" or "North Macedonia" or "Pakistan" or "Palau" or "Palestine" or "Panama" or "Papua New Guinea" or "Paraguay" or "Peru" or "Philippines" or "Rwanda" or "Saint Helena, Ascension and Tristan da Cunha" or "Saint Lucia" or "Saint Vincent and The Grenadines" or "Samoa" or "Sao Tome and Principe" or "Senegal" or "Serbia" or "Sierra Leone" or "Solomon Islands" or "Somalia" or "South Africa" or "South Sudan" or "Sri Lanka" or "Sudan" or "Suriname" or "Swaziland" or "Syrian Arab Republic" or "Syria" or "Tajikistan" or "Tanzania" or "Thailand" or "Timor-Leste" or "East Timor" or "Togo" or "Tokelau" or "Tonga" or "Tunisia" or "Turkey" or "Turkmenistan" or "Tuvalu" or "Uganda" or "Ukraine" or "Uzbekistan" or "Vanuatu" or "Venezuela" or "Vietnam" or "Wallis and Futuna Islands" or "West Bank" or "Gaza" or "Yemen" or "Zambia" or "Zimbabwe" |

(1a) AND (1b) AND (2) AND (3)

**Appendix 3.** A framework for knowledge mobilisers (Ward, 2017)

| **1-Why mobilise knowledge?** |
| --- |
| To develop local solutions to practice-based problems **(So)** |
| To develop new policies, programmes and/or recommendations **(Po)** |
| To adopt/implement clearly defined practices and policies **(Imp)** |
| To change practices and behaviours **(Ch)** |
| To produce useful research/scientific knowledge **(Kno)** |
| **2-Whose knowledge is being mobilised?** |
| Professional knowledge producers who produce empirical and/or theoretical knowledge and evidence **(KPs)** |
| Frontline practitioners and service providers responsible for delivering services to members of the public **(Pra)** |
| Members of the public acting as or on behalf of their communities and people in receipt of services **(SUs)** |
| Decision makers responsible for commissioning services an/or designing local/regional/national policies and strategies **(DMs)** |
| Product and programme developers responsible for designing, producing and/or implementing tangible products, services and programmes **(Dev)** |
| **3-What type of knowledge is being mobilised?** |
| Scientific/factual knowledge - Research findings, quality and performance data, population data and statistics, evaluation data **(Sc)** |
| Technical knowledge - Practical skills, experiences and expertise **(T)** |
| Practical wisdom - Professional judgements, values, beliefs **(Wi)** |
| **4-How is knowledge being mobilised?** |
| Making connections between knowledge stakeholders and actors by establishing and brokering relationships **(Con)** |
| Disseminating and synthesising knowledge via online databases, communication strategies, and evidence synthesis services **(Di)** |
| Facilitating interactive learning and co-production via participatory research projects and action learning sets **(Int)** |

**Appendix 4.** Overview of studies meeting the inclusion criteria

| **First author (year)** | **LMICs** | **Non-LMICs involvement** | **Aim** | **Participants** | **Investigation setting/ context** | **Mental Health problem** | **Strategy used to mobilise mental health knowledge** | **Contribution** |
| --- | --- | --- | --- | --- | --- | --- | --- | --- |
| Abayneh (2020) | Ethiopia | UK USA | To describe a ToC model for service user and caregiver involvement in a primary mental health care in rural Ethiopia. | Multiple stakeholders  >Psychiatrists >Researchers >Service users >Caregivers >Local government administrators >Health workers >Community representatives | Primary healthcare other | Mental health (no specifics) | ToC workshop | Empirical evidence |
| Abayneh (2022) | Ethiopia | UK USA | To describe the Participatory Action Research (PAR) process and explore participants’ experiences of involvement in PAR as a case study to evaluate the pilot of a model of service-user involvement in mental health system strengthening in rural primary healthcare in southern Ethiopia. | Multiple stakeholders  >Government sector office leaders >Community institution leaders (Idir leaders, Religious and faith-based) >Health professionals >Service-users >Caregivers | Primary healthcare other | Mental health (no specifics) | Participatory community consultation/ forums/ meetings/ focus groups | Empirical evidence |
| Abdulmalik  (2013) | Nigeria | Switzerland | To describe the process of adaptation and contextualization of the manual for the Nigerian health system, a system with broad similarities with those of many sub-Saharan African countries. | Multiple stakeholders  >Psychiatrists  >Social worker  >Primary care nurse  >General practitioner  >Senior technical staff of the National Primary Health Care Development Agency (the official body responsible for the standardization and development of the training programmes for primary care workers in Nigeria)  >Experienced specialists familiarised with the mhGAP programme with public health research experience | Primary healthcare other | Mental health (no specifics) | Adaptation process/  contextualization/  translation | Author reflections |
| Abrahams (2022) | South Africa | UK | This pilot study aims to evaluate the implementation outcomes of a health systems strengthening (HSS) intervention for improving the quality of care of perinatal women with CMD and experiences of domestic violence, attending public healthcare facilities in Cape Town. | Multiple stakeholders  >Pregnant women >Healthcare workers | Primary healthcare other | CMH (depression, anxiety in general) | Other (free listing, posters) | Empirical evidence |
| Adams(2020) | SierraLeone | IrelandUK | (1) To identify factors that affect the successful implementation of Community Mental Health Forums (CMHFs) and (2) Toidentify what changes participants perceived as having taken place as a result of their participation in CMHFs | Multiple stakeholders  >Mental health nurses >Traditional healers  >Imams  >Pastors  >Traditional birth attendants  >Community chiefs  >Youth leaders  >District councillors  >Community chairladies  >Mammy queens | Primary Care Mental Health Community | Mental health (no specifics) | Participatory community consultation/  forums/  meetings/  focus groups | Empirical evidence |
| Afifi  (2011) | Lebanon | Not applicable | To describe a process leading to the development of a logic model for a youth mental health promotion intervention using a participatory approach in a Palestinian refugee camp in Beirut, Lebanon | Children/ young people | Migrant/ refugee community mental health care | Mental health (no specifics) | Participatory community consultation/ forums/ meetings/ focus groups | Author reflections |
| Agarwal (2023) | India | Australia New Zealand | This study aimed to evaluate this scale-up implementation, with sub-objectives of describing the implementation and its outcomes and exploring barriers and enablers to implementation. | Multiple stakeholders  >community health workers >public health practitioners >mental health practitioners >caregivers | Primary healthcare community based | Mental health (no specifics) | Adaptation process/  contextualization/  translation | Both |
| Alonge  (2020) | EgyptIranPakistan | USAUK | To describe the SHINE network’s collaborative process for developing the pathways used at the regional and individual country levels for large-scale implementationof the SMHP. | Multiple stakeholders  >Ministry of Health  >Ministry of Education  >WHO members  >Academics  >Other | School | Mental health (no specifics) | ToC workshop | Author reflections |
| Alonzo (2023) | Peru | USA | This paper reports on the implementation of ECAPS in Lima, Peru, and the model of suicide prevention, the ¡PEDIR Program | Multiple stakeholders  >University faculty and staff >psychologists and graduate students in the psychology program >community-based mental health providers across disciplines (psychiatry, psychology, counselling, and psychiatric nursing) | Other | Mental health (no specifics) | Adaptation process/  contextualization/  translation | Author reflections |
| Appiah (2022) | South Africa Ghana | Not applicable | To describe the development of a 10‐session multicomponent positive psychology intervention, the Inspired Life Program (ILP), designed to promote mental health and reduce symptoms of depression and negative affect in rural adults in Ghana. | Adults | Rural service | Mental health (no specifics) | Participatory community consultation/ forums/ meetings/ focus groups | Author reflections |
| Atujuna  (2021) | South Africa | UKUSA | To describe the design and delivery of Khuluma, a group-based mHealth intervention, to provide psychosocial support groups to ALWH using peer-led facilitation. | Multiple stakeholders  >Adolescents living with HIV  >researchers  >health professionals | Other | Emotional distress in general | Participatory community consultation/  forums/meetings/  focus groups | Author reflections |
| Ayuso-Mateos (2019) | Ethiopia India Nepal Nigeria South Africa Uganda | UK Spain Netherlands | To describe how core elements of Emerald were implemented and aligned with the ultimate goal of strengthening mental health systems, as well as their short-term impact on practices, policies and programmes in the six partner countries | Multiple stakeholders  >policy planners >managers >patients >carers | Primary Care Mental Health other | Mental health (no specifics) | Different methods for knowledge transfer | Empirical evidence |
| Babatunde  (2022) | South Africa | Not applicable | Together with key stake-holders across multiple sectors, this study aims to (i) co-identify causal factors and potential strategies to overcome bottlenecks in one district in SA as a case study; and (ii) Co-develop a Theory of Change (ToC) for increasing access toCAMH services within the resource constraints of a remote resource-scarce district as a case study. | Multiple stakeholders  >key stakeholders from the Departments of Health (DoH), Basic Education (DBE), and Social Development (DSD)and three community-based organisations offering CAMH services in the district. >representatives from community-based organisations  >district mental health coordinator  >school directors  >service providers (e.g, nurses, clinical psychologists, occupational therapists, pharmacists, social workers, and educators) | Primary care for children/young people mental health | Mental health (no specifics) | ToC workshop | Both |
| Baheretibeb  (2022) | Ethiopia | Canada | To bridge two seemingly opposing paradigms of mental health care, traditional healing and biomedical approaches, and to enhance mental health services in communities that are distrustful of, or unfamiliar with biomedical approaches | Multiple stakeholders  >Religious healers  >Psychiatrists | Other | Mental health (no specifics) | Participatory community consultation/  forums/meetings/  focus groups | Empirical evidence |
| Bitta (2020) | Kenya | UK | To evaluate the feasibility and acceptability of the adapted mhGAP-IG in Kilifi County, Kenya, and to identify facilitators and barriers to the implementation process. The paper also aims to assess the effectiveness of the adapted guide in improving the knowledge and skills of primary healthcare workers in the identification, management, and referral of mental disorders. | Multiple stakeholders  >traditional health practitioners >primary healthcare providers >experts | Primary healthcare other | Mental health (no specifics) | Adaptation process/  contextualization/  translation | Both |
| Black  (2023) | India | USA | To showcase how 2 global mental health research teams applied HCD to develop mobile health tools, each directed at reducing treatment gaps in underserved populations. | Multiple stakeholders  >Social health activists  >trained community health workers | Rural service | CMH (depression, anxiety in general) | Participatory community consultation/  forums/meetings/  focus groups | Author reflections |
| Brooks (2021) | Indonesia | UK | To report on the development of the IMPeTUS intervention designed to improve mental health literacy amongst children and young people aged 11–15 in Java, Indonesia | Multiple stakeholders  >young people >professionals >parents >teachers >designers >academics | Other | CMH (depression, anxiety in general) | Participatory community consultation/ forums/ meetings/ focus groups | Author reflections |
| Brown (2020) | Lebanon | Netherlands Australia UK Denmark Switzerland | To outline the process and results of formative research undertaken to culturally and contextually adapt the EASE intervention to reduce psychological distress in young adolescents (age 10-14) living in Lebanon. By sharing the lessons learned, we aim to contribute to a greater understanding of necessary considerations when adapting psychosocial interventions for use in new settings | Multiple stakeholders  >Adolescents >Caregivers >Community members >Health professionals >Mental health professionals >Researchers | Other | Emotional distress in general | Adaptation process/  contextualization/  translation | Author reflections |
| Calia  (2022) | India  Syrya  Uganda  Rwanda | UK | To critically consider the nuances to achieving ethical and meaningful mental health research in three diverse settings, highlighting common strategies and approaches to achieving this. | Multiple stakeholders  >Sociotherapy participants  >Children of coal mine workers aged 6 to 23 years  >mental health researchers  >practitioners | Other | Mental health (no specifics) | Ethical issues for global mental health research | Author reflections |
| Chibanda (2016) | Zimbabwe South Africa | UK | This article describes the use of a theory of change (ToC) model to develop a brief psychoogical intervention for common mental disorders and its’ evaluation through a cluster randomized controlled trial in Zimbabwe. | Multiple stakeholders  >Policy makers from the health ministry >policy makers from city health >university lecturer >community level workers >nurse in charge >district health promoting officers >lay health workers >research team >psychiatrist >psychologists | Primary healthcare other | CMH (depression, anxiety in general) | ToC workshop | Author reflections |
| Chumo  (2022) | Kenya | Not applicable | To address the ambiguity and knowledge gap, by documenting the roles of CACs in advancing health and wellbeing in Korogocho and Viwandani informal settlements in Nairobi, Kenya. | Multiple stakeholders  >The community advisory committee working in each of the NUHDSS sites is composed of approximately 13 members. The members represent various constituencies in the community, government, local leaders/village elders, the youth, women, older persons, school administrators, healthcare providers, Faith-based organizations/ Community based organizations/Local Non-governmental organizations (FBOs/CBOs/local NGOs), Community Health Volunteers (CHVs), media/education and entertainment organizations, religious groups and people living with disabilities. | Other | Wellbeing | Participatory community consultation/  forums/meetings/  focus groups | Both |
| Coleman  (2021) | Rwanda  Peru  Mexico  Malawi | USA | To share lessons learned that can contribute to a broader understanding of effective processes for cross-site adaption of low-intensity psychological interventions (problem management plus: PM+) for common mental health conditions for people living in adversity) to real-world contexts. | Multiple stakeholders  >psychiatrists  > public health specialists  >mental health programme coordinators  >primary care nurses  >psychologists  >community health care manager  >curriculum development specialist  >expert translators  >PM+ experts  >social workers | Primary Care Mental Health Community | CMH (depression, anxiety in general) | Participatory community consultation/  forums/meetings/  focus groups | Both |
| Demissie (2024) | Ethiopia | UK | To report the process used to develop a contextualised intervention package to increase identification of depression by PHC workers in Ethiopia. | Multiple stakeholders  >PMC workers >people who were diagnosed with depression >the community advisory board >Sodo district health office representatives and PHC workers >IDEAS project investigators >mental health experts and researchers | Primary Care Mental Health Community | CMH (depression, anxiety in general) | Adaptation process/  contextualization/  translation | Author reflections |
| Fuhr  (2020) | Turkey | UK  Netherlands  Switzerland | To test the use of Theory of Change (ToC) to plan the scaling up of a low-intensity psychological intervention. | Multiple stakeholders  >Governmental officials  >mental health providers  >officials from international/national non-governmental organisations  >health researchers | Migrant/refugee community mental health care | CMH (depression, anxiety in general) | ToC workshop | Both |
| Fulone (2020) | Brazil | Not applicable | To use knowledge translation tools to support efforts that aim to improve the care that is available to deinstitutionalized people with severe mental disorders in Brazil | Multiple stakeholders  >policy-makers >HC providers >researchers in the field of public and MH >civil society organization >public defense representative | Secondary Care | Other disorder specific (bipolar, psychosis) | Participatory community consultation/ forums/ meetings/ focus groups | Empirical evidence |
| Gellatly (2022) | India | UK USA | To provide a qualitative analysis of collaborative efforts to build a school-based intervention for multiple common adolescent mental health difficulties in India | Multiple stakeholders  >Mental health providers employed by Sangath, a non-governmental, non-profit organization conducting research and providing psychosocial services across India | School | CMH (depression, anxiety in general) | Participatory community consultation/ forums/ meetings/ focus groups | Both |
| Giebel  (2024) | Colombia and other LMICs | UK  Australia | To explore the key lessons for developing, implementing, and evaluating community-based mental health and well-being interventions in LMICs, with an additional focus on older adults. | Multiple stakeholders  >International experts in designing, researching and implementing mental health interventions in LMICs including multidisciplinary academics, analysts and clinicians | University | Mental health (no specifics) | Participatory community consultation/  forums/meetings/  focus groups | Empirical evidence |
| Gigaba (2023) | South Africa | USA | To describe the implementation of an intervention called the Mental health INTegration Programme (MhINT), which aimed to introduce an evidence-based collaborative care package for depression into the PHC platform and simultaneously strengthen the health system to embed the intervention. | Multiple stakeholders  >multidisciplinary Continuous Quality Improvement (CQI) teams | Primary healthcare other | CMH (depression, anxiety in general) | Adaptation process/  contextualization/  translation | Author reflections |
| Giusto (2023) | Kenya | USA | To adapt a PST intervention for peer-delivery to treat youth presenting to a community center in western Kenya and evaluate whether peer counsellors can be trained on the intervention | Children/young people 18-24 year olds | Other | CMH (depression, anxiety in general) | Adaptation process/  contextualization/  translation | Author reflections |
| Greene  (2022) | EcuadorPanama | USADenmark | To describe the community-based participatory intervention design process, as well as the results of formative research describing and connecting the needs of migrant women in Ecuador and Panama to intervention and implementation strategies that fit those needs | Multiple stakeholders  >Adult females who had migrated to the study community  >key informants who were knowledgeable about MH and issues facing migrant women (e.g. community members, leaders, police, women's shelter workers etc). | Migrant/ refugee community mental health care | Mental health (no specifics) | Participatory community consultation/  forums/meetings/  focus groups | Author reflections |
| Hafting (2022) | Cambodia | Norway | To describe experiences and practical concerns from the perspective of the local staff of a transcultural program in capacity-building in child mental health in LMIC. | Multiple stakeholders  >staff members of Caritas-CCAMH who had taken part in the program for at least 2 years >nurses > psychologists >special educator > social worker >speech therapist >occupation therapist >art therapist >program administrator | Primary care for children/ young people mental health | Mental health (no specifics) | Adaptation process/  contextualization/  translation | Empirical evidence |
| Hailemariam  (2015) | Ethiopia | UK | To report the process of implementing the ToC approach in developing mental health care plan. | Multiple stakeholders  >federal and local policy makers  >mental health experts and researchers  > community representatives (e.g., district administration, police, health, representatives of faith and traditional healers, women’s and children’s affairs and youth associations)  >mental health professionals including clinical psychologists, public health professionals, social workers and psychiatrists (in the policy makers’ group). | Other | Mental health (no specifics) | ToC workshop | Empirical evidence |
| Hamdani  (2021) | Pakistan | UKUSA | To develop a model for scale-up of school based mental health services in public schools of Pakistan. | Multiple stakeholders  >policy makers  >representatives from district education planners and officers  >mental health specialists  >international partners (representatives from WHO country and regional offices)  >international child and adolescent mental health experts and iNGO working on child and adolescent mental health  >national partners (e.g. NGOs, psychologists, psychiatrists)  >representatives from education department, health department, IoP-WCC >researchers  >head teachers and teachers | School | Mental health (no specifics) | ToC workshop | Empirical evidence |
| Hameed  (2020) | India | Not applicable | To explore perspectivesof stakeholders (college youth and faculty/ administrators) about felt needs, potential content, feasibility, and receptivity of a PYD program for MHP. It formed a part of alarger research project on development and pilot testing ofa PYD program for Indian college-going youth. | Multiple stakeholders  >college students  >college faculty administrators | University | Wellbeing | Participatory community consultation/  forums/meetings/  focus groups | Author reflections |
| Haroz (2019) | Ukraine | USA | To: 1) adapt an IS measure for a specific context; 2) develop vignettes depicting existing and hypothetical mental health care services to use as criteria in a testing study; and 3) test the reliability and validity of the measure among a sample of mental health service consumers in Ukraine using the vignettes. | Multiple stakeholders  >veterans >Internally Displaced Persons | Other | Mental health (no specifics) | Participatory community consultation/ forums/ meetings/ focus groups | Author reflections |
| Hoven (2008) | Armenia Azerbaijan Brazil China Egypt Georgia Israel Uganda | Russia | To develop and test a feasible strategy to promote child mental health awareness, one that could inform and guide the implementation of such a programme worldwide. | Multiple stakeholders  >students >parents >teachers | School | Mental health (no specifics) | Other (free listing, posters) | Both |
| Kaysen  (2013) | Kurdistan | USA | To culturally adapt and implement Cognitive Processing Therapy (CPT) for use in Kurdistan. | Mental Health professionals (i.e.,  Community Mental Health Workers and clinical supervisor) | Primary Care Mental Health Community | Mental health (no specifics) | Adaptation process/  contextualization/  translation | Both |
| Khan (2023) | Pakistan | UK | To illustrate the process of adapting mhGAP materials to develop psychosocial support capacity-building resources using the HCD framework for supporting maternity patients in the labour room | Multiple stakeholders  >Clinical maternity staff of the labour room (obstetrician/ gynaecologist, midwife, lady health visitor, nurse, and technician) >non-clinical maternity staff (traditional birth attendant, maid/ sweeper, security guard) | Primary healthcare hospital | Wellbeing | Adaptation process/  contextualization/  translation | Author reflections |
| Kohrt (2022) | Guatemala | USA | To: 1) identify areas of the THP/PENSA program (low intensity intervention) that require adaptation prior to implementation within a Tz'utujil Mayan community in Guatemala, and 2) develop a training protocol for local community health workers that is centered in their culture, context, and worldview. | Multiple stakeholders  >mothers >community health workers >psychologist >NGO staff >Mayan artist | Primary healthcare community based | CMH (depression, anxiety in general) | Adaptation process/  contextualization/  translation | Both |
| Laurenzi  (2024) | Nepal  South Africa | UK  Switzerland  USA | To detail the multi-stage development of the HASHTAG intervention. | Multiple stakeholders  >Adolescents  >caregivers/parents  >teachers  >key community stakeholders | School | Mental health (no specifics) | Participatory community consultation/  forums/meetings/  focus groups | Author reflections |
| Le (2023) | Kenya Tanzania | UK | To document how the Mothers and Babies Course (MBC), a cognitive–behavioral (CBT) intervention for perinatal mothers at risk for depression was adapted to fit the contexts of rural pregnant women and mothers of young children in Kenya and Tanzania using the Framework for Reporting Adaptations and Modifications-Enhanced (FRAME) | Multiple stakeholders  >individuals from Catholic Relief Services >care group volunteers >mothers >case managers | Primary Care Mental Health Community | CMH (depression, anxiety in general) | Adaptation process/  contextualization/  translation | Both |
| Li (2023) | China | Not applicable | To explore the cultural fitness of TF-CBT in China and documenting the adaptation process | Multiple stakeholders  >mental health practitioners >caregivers >school staff >children | School | CMH Disorder specific (OCD, PTSD) | Adaptation process/  contextualization/  translation | Author reflections |
| Liem  (2022) | China | Germany | Using an implementation science framework, this study aimed to evaluate stakeholders’ perspectives on the implementation of Kumusta Kabayan, a mobile phone-based mental health app, for OFWs in Macao | Multiple stakeholders  >Overseas Filipino Workers team members  >local non-governmental organization (NGO) staff members  >Overseas Filipino Workers in Macao | Other | Mental health (no specifics) | Adaptation process/  contextualization/  translation | Author reflections |
| Lovero (2022) | Mozambique | USA | To employ Implementation Mapping to develop a multilevel strategy for integrating adolescent depression services within primary care clinics of Maputo, Mozambique | Multiple stakeholders  >Key informants >mental health specialists >primary care providers >medicine technicians >nurses >other health professionals (e.g., sexual and reproductive health counsellors, physician) | Primary care for children/ young people mental health | Mental health (no specifics) | Participatory community consultation/ forums/ meetings/ focus groups | Author reflections |
| MacDougall (2021) | Kenya | Canada | This paper outlines the development processes and evaluation of the proof of concept for the Community REcovery Achieved Through Entrepreneurship (CREATE) strategy in Kenya. | Multiple stakeholders  >Community dwelling adults living with serious mental illness >Family members  >Community members from mental health | Primary Care Mental Health Community | Mental health (no specifics) | Participatory community consultation/ forums/ meetings/ focus groups | Both |
| Maddock(2023) | Cambodia | UK | To describe a research priority setting workshop in order to: 1) identify the key issues which may limit the extent to which people with mental health issues and disabilities receive appropriate support, 2) identify the servicesand interventions currently available, 3) identify the keyservices and interventions that need to be implemented,and 4) to develop a list of the most important researchpriorities in mental health, which would inform thedevelopment of effective and sustainable strategies for prevention and intervention in Cambodia. | Multiple stakeholders  >government officials (1 junior government minister and 1 deputy toa government minister)  >disability professionals (e.g., social workers, prosthetists  >non-professional workers  >disabilityadvocacy professionals  >mental health professionals (e.g., social workers, adultpsychiatrists, psychologists, occupational therapists, child and adolescent psychiatrist)  >mental health academics (e.g., psychology, social work)  >disability academics (e.g., social work) | Other | Mental health (no specifics) | Participatory community consultation/  forums/meetings/  focus groups | Author reflections |
| Magidson (2015) | Iraq | Not applicable | This study describes the adaptation of the community health workers (CHW) delivered behavioral activation treatment for depression (BATD). | Multiple stakeholders  >CHWs >a study psychiatrist >CHW clinical supervisor | Primary healthcare other | CMH Disorder specific (OCD, PTSD) | Adaptation process/ contextualization/ translation | Author reflections |
| Matsea  (2022) | South Africa | Not applicable | To present a framework for social workers to developand implement a community-based programme (CBP) in rural settings. | Multiple stakeholders  >health professionals  >families  >community members | Rural service | Mental health (no specifics) | Participatory community consultation/ forums/meetings/ focus groups | Author reflections |
| Maulik (2016) | India | UK Australia | To develop and evaluate the feasibility, acceptability and preliminary effectiveness of a multifaceted primary healthcare worker. | Service users | Rural service | CMH (depression, anxiety in general) | Other (free listing, posters) | Author reflections |
| Memiah (2022) | Kenya | USA | To identify mental health challenges faced by adolescents and young people in Kenya, develop practical recommendations to mitigate these issues, and reduce the mental health burden among this population. | Multiple stakeholders  >adolescents and young people >MoH Officials >Researchers >Program implementers | Primary care for children/ young people mental health | CMH (depression, anxiety in general) | Participatory community consultation/ forums/ meetings/ focus groups | Both |
| Mukherjee  (2024) | India | UK  Canada  Australia | 1. To obtain information on the local context (includingkey stakeholders), factors influencing the uptake ofmental health services and common stressors amongadolescents living in the study area.2. To assess the feasibility of rolling out a community based anti-stigma campaign and the acceptability ofanti-stigma IEC content among adolescents, and3. To assess the feasibility of rolling out the mHealthcomponent of the intervention and acceptability of the EDSS among ASHAs and UPHC doctors. | Multiple stakeholders  >adolescents  >parents  >doctors  >community health workers  >subject matter experts | Other | CMH (depression, anxiety in general) | ToC workshop | Author reflections |
| Murphy (2024) | Vietnam | Canada Australia | To explore factors that have contributed to policy development as well  as challenges faced in the ongoing mental health policy and practice context, including the COVID-19 pandemic. | Multiple stakeholders  >Researchers >government representatives | Other | CMH (depression, anxiety in general) | Knowledge translation approach to research policy collaboration | Both |
| Murray  (2013) | Zambia | USA | To select and culturally adapt an intervention for CYP. | Multiple stakeholders  > individuals from local academic institutions  >organizations working on child mental health issues  >mothers from the community  >lay counsellors | Other | CMH Disorder specific (OCD, PTSD) | Participatory community consultation/ forums/meetings/ focus groups | Author reflections |
| Mutahi (2024) | Kenya | Not applicable | To document the processes around the design and implementation of this innovative intervention (Bridging the Gaps (BTG) safe space health, life, financial, and gender norms) and present a summary of lessons learned. | Multiple stakeholders  >out-of-school adolescents, their parents, and mentors >people working in the private, public, and NGO sectors selected based on their experience of working in mental health and/ or adolescent health. | Primary Care Mental Health Community | Mental health (no specifics) | Adaptation process/ contextualization/  translation | Author reflections |
| Mutiso, Musyimi  (2018) | Kenya | Netherlands Canada | To describe a model for a stepwise approach for implementation of mhGAP-IG in a rural Kenyan setting using existing formal and informal community resources and health systems. | Multiple stakeholders  >local administrators >village heads as gate keepers to the community >parents >health providers >school teachers >policy makers >children | School | Wellbeing | Adaptation process/  contextualization/  translation | Empirical evidence |
| Mutiso, Gitonga  (2018) | Kenya | USA | This study aims at documenting the process of implementing "Kenya Integrated intervention model for Dialogue and Screening to promote children's mental wellbeing (KIDS)", with emphasis on activities, experiences, challenges and lessons learnt through the process that can inform improvement in Kenya and other social-economically and culturally similar settings. | Multiple stakeholders  >local administrators  >village heads as gate keepers to the community  >parents  >health providers  >school teachers  >policy makers  >children | School | Mental health (no specifics) | Adaptation process/  contextualization/  translation | Author reflections |
| Naslund  (2021) | East Asia and the Pacific (PRISM); Latin America and the Caribbean (DIADA); the Middle East and North Africa (SHINE); South Asia (ESSENCE and SPIRIT); and Sub-Saharan Africa | USA | To identify key challenges and recommendations for advancing global mental health implementation research in low-and middle-income countries (LMICs) | Multiple stakeholders  >researchers  >local stakeholders  >end-users of the program | Primary healthcare other | Mental health (no specifics) | Participatory community consultation/  forums/meetings/  focus groups | Empirical evidence |
| Nguyen (2023) | Ukraine | USA Ireland | To describe the develop- ment and feasibility testing of CETA Psychosocial Support (CPSS), a brief psychosocial prevention and referral program for Ukrainian veterans and their families. | Multiple stakeholders  >Veterans and their families >experts (e.g., social workers, volunteers, lawyers, and heads of charitable foundations) | Primary Care Mental Health Community | Mental health (no specifics) | Participatory community consultation/ forums/ meetings/ focus groups | Author reflections |
| O'Donnell (2022) | Colombia Mexico  Peru | UKNetherlandsGermanySpain | To describe the process of development and cultural adaptation of the SCALA clinical intervention and training package in Latin America. | Multiple stakeholders  >patients  >healthcare providers  >community leaders | Primary healthcare other | CMH Disorder specific (OCD, PTSD) | Adaptation process/ contextualization/  translation | Author reflections |
| Orengo-Aguayo (2020) | El Salvador | USA | To describe the implementation of Trauma-Focused Cognitive Behavioral Therapy (TF-CBT) in 3 separate low-resourced settings (rural South Carolina, Puerto Rico, and El Salvador) utilizing the Exploration, Preparation, Implementation, and Sustainment (EPIS) framework and guided by a community-based participatory research framework. | Multiple stakeholders   >policymakers, including USAID, the El Salvador Ministry of Education,  >mental health providers >nonprofit agencies | School | CMH Disorder specific (OCD, PTSD) | Adaptation process/ contextualization/  translation | Author reflections |
| Passchier  (2019) | South Africa | USA  UK  Netherlands | To conduct formative health systems research on the implementation of routine depression screening using adigital tool – Mood in Retroviral Positive Individuals Application Monitoring (MIR + IAM) – in an HIV primary caresetting in South Africa. | Multiple stakeholders  >experts  >local stakeholders  >HIV health care service providers  >working professional  >academic researchers in the fields of mental health, HIV, health systems or digital technology | Primary healthcare other | CMH Disorder specific (OCD, PTSD) | ToC workshop | Author reflections |
| Perera (2020) | Colombia | Ireland Denmark | To describe how cultural adaptations can be implemented for psychological interventions within humanitarian contexts in a systematic and timely way. This process was illustrated through its application to the cultural adaptation of the World Health Organization (WHO)’s Problem Management Plus (PM+) intervention to Venezuelan migrants and refugees and Colombian Internally Displaced Persons (IDPs) living in Saravena, Colombia. | Multiple stakeholders  >MHPSS specialists >programme implementers >community member.  >Colombian and a Venezuelan psychologist, both based in academic institutions | Migrant/ refugee community mental health care | CMH (depression, anxiety in general) | Adaptation process/ contextualization/ translation | Author reflections |
| Petersen  (2022) | South Africa | UK | To describe the intervention tested in the trials (collaborative care model) and better understand how context impacted on implementation of the package and service delivery outcomes across the two district sites. | Multiple stakeholders  >service managers  >service providers  >patients  >caregivers | Primary Care Mental Health other | CMH (depression, anxiety in general) | Adaptation process/ contextualization/ translation | Author reflections |
| Premji (2021) | China | Canada | To engage multiple stakeholders to assess the integration of perinatal depression screening and iCBT within a flexible framework that would consider context, ensure that mental healthcare is responsive to women’s needs, and promote implementation outcomes of acceptance, reach, scope, and scale. | Multiple stakeholders  >healthcare providers (physicians, nurses, and midwives) >pregnant and postpartum women and their families >policy makers | Other | CMH Disorder specific (OCD, PTSD) | Participatory community consultation/ forums/ meetings/ focus groups | Empirical evidence |
| Rai  (2023) | Nepal  Ethiopia  Uganda | USA  UK | To provide a guiding framework for using PhotoVoice to collaborate with PWLE in anti-stigma and mental healthcare strengthening programs using recovery narratives in low-resource settings.Discuss the potential benefits and challenges of using PhotoVoice to collaborate with PWLE and provide a way forward for future research using this method. | Multiple stakeholders  >People with lived experience of mental illness  >caregivers  >trainers  >counsellors  >audiences of the recovery stories. | Primary Care Mental Health Community | Mental health (no specifics) | Participatory community consultation/  forums/meetings/  focus groups | Author reflections |
| Rivera (2008) | Peru | Spain USA | To describe and analyse the first comprehensive case study of the application of the IASC Guidelines on Mental Health and Psychosocial Support by Medicos del Mundo- Spain after the August 2007 earthquake in Peru. | Multiple stakeholders  >health personnel >community leaders  >individuals | Other | Mental health (no specifics) | Participatory community consultation/ forums/ meetings/ focus groups | Both |
| Sangraula  (2021) | Nepal | USA  Switzerland  Australia | To provide a transparent, thorough, and prescriptive framework to guide rapid and systematic adaptations of evidence-based psychological interventions in LMICs and humanitarian settings. | Multiple stakeholders  >female community health volunteers  >leaders  >health workers  >community members  >field staff  >intervention participants  >participants’ families  >other key community stakeholders | Other | CMH (depression, anxiety in general) | Adaptation process/ contextualization/  translation | Both |
| Sapag (2016) | Mexico Nicaragua Chile | Canada | To identify evaluation needs, as well as potential implementation challenges and opportunities, as perceived by key healthcare leaders and professionals regarding the development of an evaluation framework in three CMHC systems located in Mexico, Nicaragua and Chile. Aimed to develop an evaluation framework of CMHC at the district or municipal level in Latin America. | Multiple stakeholders  >decision-makers >front-line clinicians >other stakeholders through | Other | CMH (depression, anxiety in general) | Participatory community consultation/ forums/ meetings/ focus groups | Empirical evidence |
| Shidhaye  (2019) | India | UK | To provide quantitative measures of outputs related to implementation processes, describe the role of contextual factors that facilitated and impeded implementation processes, and discuss what has been learned from the MHCP implementation. | Multiple stakeholders  >CHC medical officers  >Community health workers  >District mental health programme psychiatrist  >PRIME team members  >PRIME core team  >PRIME case managers | Rural service | CMH (depression, anxiety in general) | Participatory community consultation/ forums/meetings/ focus groups | Empirical evidence |
| Singh (2021) | Tanzania | USA UK | To develop a culturally appropriate and contextually relevant intervention that addresses the mental health concerns of Burundian refugee adolescents living in refugee camps. The study also seeks to understand the mental health concerns and preferences for seeking support from the perspective of adolescents, caregivers, and other stakeholders. | Multiple stakeholders  >Young people >caregivers (parents, teachers, community leaders and health or protection workers) >mental health professionals | Refugee camps | CMH (depression, anxiety in general) | Adaptation process/ contextualization/ translation | Author reflections |
| Sit  (2022) | China | Germany  USA | To assess feasibility of recruitment and of delivery of Step-by-Step in a University setting, to assess acceptability of theintervention, and to examine potential effectiveness. | University Students aged 18 or more | University | CMH (depression, anxiety in general) | Participatory community consultation/ forums/meetings/ focus groups | Empirical evidence |
| Spagnolo  (2020) | Tunisia Coˆte d’Ivoire | Canada | Tunisia Project only To improve continuing mental health training for primary care physicians healthcare professionals.  To describe the knowledge translation strategies in two projects and share lessons learned about knowledge sharing and uptake.  Data extraction is focus on the project conducted in Tunisia which is the only one focused on mental health | Multiple stakeholders  >primary care physicians >the Presidents of the Committee for Mental Health Promotion >the Technical Committee for Suicide Prevention >the Tunisia WHO office Health Systems Advisor | Primary Care Mental Health Community | Mental health (no specifics) | Different strategies for knowledge transfer | Author reflections |
| Ssebunnya  (2021) | Uganda | USA | To describe the process of developing a model for integrating the management of depression in HIV care in Uganda. Theory of Change (ToC) methodology was used to guide the process of developing the model. | Multiple stakeholders  >district health service managers  >members from the political leadership and administrative officers  >health facility managers  >primary health care service providers  >district HIV focal person  >staff from NGOs involved in health, mental health specialists  >religious leaders  >Community Health Workers  >HIV care service users/expert clients. | Other | CMH Disorder specific (OCD, PTSD) | ToC workshop | Both |
| Tinago (2021) | Zimbabwe | USA | To develop a community-based peer support intervention to mitigate social isolation and stigma of adolescent motherhood and improve mental health of adolescent mothers in Harare, Zimbabwe. | Multiple stakeholders  >adolescent mothers aged 14-18 years >community health workers >teachers and family members of adolescent mothers >health officials >religious leaders | Other | Mental health (no specifics) | Participatory community consultation/ forums/ meetings/ focus groups | Empirical evidence |
| Triplett  (2023) | Kenya | USA | To evaluate the effects of sharing co-developedimplementation guidelines in a brief educational outreach visit on the acceptability, feasibility, and usability of mobile phone supervision for lay counsellors in Kenya | Multiple stakeholders  >counsellors  >supervisors | School | CMH Disorder specific (OCD, PTSD) | Participatory community consultation/ forums/meetings/ focus groups | Author reflections |
| vanderBoor (2024) | Uganda | UK Denmark Netherlands Germany | To use ToC to summarize pathways to the implementation and maintenance of the CHANGE intervention for refugees in the context of a refugee settlement in northern Uganda. | Multiple stakeholders  >Health and education professionals >community representatives >third sector staff >refugee leaders >religious leaders >programme staff | Migrant/ refugee community mental health care | Emotional distress in general | ToC workshop | Both |
| Wasil (2020) | India | USA | This paper describes the selection, adaption, and pilot testing of three single-session interventions (SSIs) for adolescents aimed to improve youth well-being and mental health in Pune, a city in the Indian state of Maharashtra. | Multiple stakeholders  >teachers >educators >administrators from high schools >Students from grades 7 through 12 | School | Mental health (no specifics) | Participatory community consultation/ forums/ meetings/ focus groups | Author reflections |
| Wieling  (2017) | Uganda | USA Germany | To describe the initial development of the program and to assess community-level need and desire for a parenting intervention. | Multiple stakeholders  >parents  >children aged 5-12  >counsellors | Other | CMH Disorder specific (OCD, PTSD) | Participatory community consultation/ forums/meetings/ focus groups | Both |

**Appendix 5.** Quality appraisal performed using the Mixed Methods Appraisal Tool (MMAT)^30^

| **First Author**  **(year)** | **Screening Questions** | | **Qualitative Studies Questions** | | | | | **Quantitative Descriptive Studies Questions** | | | | | **Mixed Methods Studies Questions** | | | | | **Notes** |
| --- | --- | --- | --- | --- | --- | --- | --- | --- | --- | --- | --- | --- | --- | --- | --- | --- | --- | --- |
|  | **1** | **2** | **1** | **2** | **3** | **4** | **5** | **1** | **2** | **3** | **4** | **5** | **1** | **2** | **3** | **4** | **5** |  |
| Abayneh (2020) | Yes | Yes | Yes | Yes | Yes | Yes | Yes |  |  |  |  |  |  |  |  |  |  |  |
| Abayneh (2022) | Yes | Yes | Yes | Yes | Yes | Yes | Yes |  |  |  |  |  |  |  |  |  |  |  |
| Abrahams (2022) | Yes | Yes |  |  |  |  |  |  |  |  |  |  | Yes | Yes | Yes | Can't tell | Yes |  |
| Adams (2020) | Yes | Yes | Yes | Yes | Yes | Yes | Yes |  |  |  |  |  |  |  |  |  |  |  |
| Agarwal (2023) | Yes | Yes |  |  |  |  |  |  |  |  |  |  | No | Yes | Yes | No | No |  |
| Ayuso-Mateos (2019) | Yes | Yes |  |  |  |  |  |  |  |  |  |  |  |  |  |  |  | This is descriptive study, no choice for method. |
| Babatunde (2022) | Yes | Yes | Yes | Yes | Yes | Yes | Yes |  |  |  |  |  |  |  |  |  |  |  |
| Baheretibeb (2022) | Yes | Yes |  |  |  |  |  |  |  |  |  |  |  |  |  |  |  | This study is a case study, no choice for method. |
| Bitta (2020) | Yes | Yes | Yes | Yes | Yes | Yes | Yes |  |  |  |  |  |  |  |  |  |  | Focus on the situational analysis, stakeholder engagement, and local adaptation process |
| Chumo (2022) | Yes | Yes | Yes | Yes | Yes | Yes | Yes |  |  |  |  |  |  |  |  |  |  |  |
| Coleman (2021) | Yes | Yes |  |  |  |  |  |  |  |  |  |  |  |  |  |  |  | This is descriptive study, no choice for method. |
| Fuhr (2020) | Yes | Yes | Yes | Yes | Can't tell | Can't tell | Can't tell |  |  |  |  |  |  |  |  |  |  |  |
| Fulone (2020) | Yes | Yes | Yes | Yes | Yes | Can't tell | Yes |  |  |  |  |  |  |  |  |  |  |  |
| Gellatly (2022) | Yes | Yes | Yes | No | Yes | Yes | Yes |  |  |  |  |  |  |  |  |  |  |  |
| Giebel  (2024) | Yes | Yes | Yes | Yes | Yes | Yes | Yes |  |  |  |  |  |  |  |  |  |  |  |
| Hafting (2022) | Yes | Yes | Yes | Yes | Yes | Yes | Yes |  |  |  |  |  |  |  |  |  |  |  |
| Hailemariam (2015) | Yes | Yes | Yes | Yes | Yes | Yes | Yes |  |  |  |  |  |  |  |  |  |  |  |
| Hamdani (2021) | Yes | Yes | Yes | Yes | Yes | Yes | Yes |  |  |  |  |  |  |  |  |  |  |  |
| Hoven (2008) | Yes | Yes |  |  |  |  |  | Can't tell | Can't tell | Yes | Can't tell | Yes |  |  |  |  |  |  |
| Kaysen (2013) | No | Can't tell |  |  |  |  |  |  |  |  |  |  |  |  |  |  |  |  |
| Kohrt (2022) | Yes | Yes |  |  |  |  |  |  |  |  |  |  | Yes | Yes | Yes | Can't tell | No |  |
| Le (2023) | Yes | Yes |  |  |  |  |  |  |  |  |  |  | Yes | Yes | Can't tell | Yes | No |  |
| MacDougall (2021) | Can't tell | Yes | Yes | Yes | Yes | Yes | Yes |  |  |  |  |  |  |  |  |  |  | This is evaluation of project (CREATE) |
| Memiah (2022) | Yes | Yes |  |  |  |  |  |  |  |  |  |  | Yes | Yes | Yes | Yes | Yes |  |
| Murphy (2024) | Yes | Yes | Yes | Yes | Yes | Yes | Yes |  |  |  |  |  |  |  |  |  |  |  |
| Mutiso, Musyimi (2018) | Yes | Yes |  |  |  |  |  |  |  |  |  |  |  |  |  |  |  | This study is a case study, no choice for method. |
| Naslund (2021) | Yes | Yes |  |  |  |  |  |  |  |  |  |  | Yes | Yes | Can't tell | Can't tell | Can't tell |  |
| Premji (2021) | Yes | Yes | Yes | Can't tell | Yes | Yes | Yes |  |  |  |  |  |  |  |  |  |  |  |
| Rivera (2008) | Yes | Yes | Yes | Yes | Can't tell | Yes | Yes |  |  |  |  |  |  |  |  |  |  |  |
| Sangraula  (2021) | Yes | Yes |  |  |  |  |  |  |  |  |  |  | Yes | Yes | Yes | Can't tell | Yes |  |
| Sapag (2016) | Yes | Yes |  |  |  |  |  |  |  |  |  |  | Yes | Can't tell | Can't tell | Yes | Yes |  |
| Shidhaye (2019) | Yes | Yes |  |  |  |  |  |  |  |  |  |  | Yes | Yes | Yes | Yes | Yes |  |
| Sit (2022) | Yes | Yes |  |  |  |  |  |  |  |  |  |  | Yes | Yes | Can't tell | Can't tell | Yes |  |
| Ssebunnya (2021) | Yes | Yes | Yes | Yes | Yes | Yes | Yes |  |  |  |  |  |  |  |  |  |  |  |
| Tinago (2021) | Yes | Yes | Yes | Yes | Yes | Yes | Yes |  |  |  |  |  |  |  |  |  |  |  |
| Van der Boor (2024) | Yes | Yes | Yes | No | Yes | Yes | Yes |  |  |  |  |  |  |  |  |  |  |  |
| Wieling (2017) | Yes | Yes | Yes | Yes | Yes | Yes | Yes |  |  |  |  |  |  |  |  |  |  |  |

**Appendix 6.** Framework for knowledge mobilisers implemented for included papers

| First author (year) | **1-Why mobilise knowledge?** | | | | | **2-Whose knowledge is  being mobilised?** | | | | | **3-What type of knowledge is being mobilised?** | | | **4-How is knowledge being mobilised?** | | |
| --- | --- | --- | --- | --- | --- | --- | --- | --- | --- | --- | --- | --- | --- | --- | --- | --- |
|  | **1.1** | **1.2** | **1.3** | **1.4** | **1.5** | **2.1** | **2.2** | **2.3** | **2.4** | **2.5** | **3.1** | **3.2** | **3.3** | **4.1** | **4.2** | **4.3** |
| Abayneh (2020) | Yes | No | Yes | No | No | Yes | Yes | Yes | Yes | No | No | Yes | No | Yes | No | Yes |
| Abayneh (2022) | No | No | No | No | Yes | Yes | Yes | Yes | Yes | No | No | Yes | No | Yes | No | Yes |
| Abdulmalik (2013) | No | No | Yes | Yes | No | No | Yes | No | No | No | No | Yes | No | Yes | No | Yes |
| Abrahams (2022) | No | No | Yes | No | No | Yes | No | No | No | No | No | Yes | No | Yes | No | No |
| Adams (2020) | No | No | Yes | Yes | No | No | Yes | Yes | No | No | No | Yes | No | No | No | Yes |
| Afifi (2011) | Yes | No | Yes | No | No | No | Yes | Yes | Yes | No | No | Yes | Yes | No | No | Yes |
| Agarwal (2023) | No | Yes | No | No | No | No | Yes | Yes | No | No | No | Yes | Yes | Yes | No | Yes |
| Alonge (2020) | No | No | Yes | No | No | No | Yes | Yes | Yes | No | No | Yes | Yes | Yes | No | Yes |
| Alonzo (2023) | Yes | Yes | Yes | Yes | No | No | Yes | No | Yes | Yes | Yes | Yes | Yes | Yes | No | Yes |
| Appiah (2022) | Yes | No | No | No | No | No | No | Yes | No | No | No | No | Yes | No | No | Yes |
| Atujuna (202X) | Yes | No | Yes | No | No | Yes | Yes | Yes | No | No | No | Yes | Yes | No | No | Yes |
| Ayuso-Mateos (2019) | No | No | Yes | No | No | Yes | Yes | Yes | Yes | Yes | Yes | Yes | No | Yes | Yes | Yes |
| Babatunde (2022) | No | No | No | Yes | Yes | No | Yes | No | Yes | No | No | Yes | Yes | Yes | No | Yes |
| Baheretibeb (2022) | No | No | No | Yes | Yes | No | Yes | No | No | No | No | Yes | Yes | Yes | No | Yes |
| Bitta (2020) | No | No | Yes | Yes | Yes | No | Yes | No | Yes | No | No | Yes | Yes | Yes | No | Yes |
| Black (2023) | Yes | No | No | Yes | No | No | No | Yes | No | No | No | No | Yes | Yes | No | Yes |
| Brooks (2021) | Yes | No | No | No | No | Yes | Yes | Yes | No | Yes | No | Yes | No | No | No | Yes |
| Brown (2020) | No | No | Yes | No | No | Yes | Yes | Yes | No | No | No | Yes | Yes | No | No | Yes |
| Calia (2022) | No | No | No | Yes | No | Yes | No | No | No | No | No | No | Yes | No | Yes | No |
| Chibanda (2016) | Yes | Yes | Yes | No | Yes | Yes | Yes | Yes | Yes | No | Yes | Yes | Yes | Yes | No | Yes |
| Chumo (2022) | Yes | Yes | Yes | Yes | Yes | Yes | Yes | Yes | Yes | Yes | No | Yes | Yes | Yes | Yes | Yes |
| Coleman (2021) | No | Yes | No | No | No | Yes | Yes | No | Yes | No | Yes | Yes | Yes | Yes | No | No |
| Demissie (2024) | Yes | Yes | No | No | Yes | Yes | Yes | Yes | Yes | No | Yes | Yes | Yes | Yes | Yes | Yes |
| Fuhr (2020) | No | No | Yes | No | No | Yes | Yes | No | Yes | Yes | No | Yes | Yes | Yes | No | No |
| Fulone (2020) | Yes | No | Yes | No | No | Yes | Yes | No | Yes | No | No | Yes | Yes | Yes | Yes | Yes |
| Gellatly (2022) | Yes | Yes | No | No | Yes | No | Yes | No | No | No | No | Yes | Yes | No | No | Yes |
| Giebel (2024) | Yes | Yes | Yes | Yes | No | No | Yes | Yes | No | No | No | Yes | Yes | No | No | Yes |
| Gigaba (2023) | Yes | No | Yes | Yes | No | No | Yes | Yes | Yes | No | Yes | Yes | No | Yes | No | Yes |
| Giusto (2023) | Yes | Yes | No | No | Yes | No | No | Yes | No | No | No | Yes | No | No | No | Yes |
| Greene (2022) | Yes | Yes | Yes | Yes | Yes | Yes | Yes | Yes | Yes | Yes | No | Yes | Yes | No | No | Yes |
| Hafting (2022) | No | No | Yes | No | No | No | Yes | No | No | No | No | Yes | No | No | No | Yes |
| Hailemariam (2015) | No | Yes | Yes | No | No | Yes | Yes | No | Yes | Yes | Yes | Yes | Yes | Yes | Yes | Yes |
| Hamdani (2021) | Yes | No | Yes | No | No | Yes | Yes | Yes | Yes | Yes | No | Yes | Yes | No | No | Yes |
| Hameed (2020) | Yes | Yes | No | No | Yes | No | No | Yes | Yes | Yes | No | Yes | Yes | Yes | No | Yes |
| Haroz (2019) | No | No | Yes | No | No |  |  |  |  |  | No | Yes | No |  |  |  |
| Hoven (2008) | No | No | No | Yes | No | Yes | Yes | Yes | Yes | No | No | Yes | No | No | No | Yes |
| Kaysen (2013) | No | No | Yes | No | No | Yes | No | No | No | No | No | Yes | No | No | No | Yes |
| Khan (2023) | No | Yes | No | Yes | No | No | Yes | No | No | No | Yes | Yes | Yes | No | No | Yes |
| Kohrt (2022) | Yes | Yes | Yes | Yes | Yes | Yes | Yes | Yes | No | Yes | Yes | Yes | Yes | Yes | Yes | Yes |
| Laurenzi (2024) | Yes | No | No | No | No | Yes | Yes | Yes | Yes | No | Yes | Yes | Yes | No | No | Yes |
| Le (2023) | No | Yes | No | No | No | No | Yes | Yes | Yes | Yes | Yes | Yes | Yes | Yes | No | Yes |
| Li (2023) | No | Yes | No | No | Yes | Yes | Yes | Yes | Yes | No | Yes | Yes | Yes | No | No | Yes |
| Liem (2022) | No | No | Yes | No | No | No | Yes | Yes | No | No | No | Yes | No |  |  |  |
| Lovero (2022) | No | No | Yes | No | No | No | Yes | No | No | No | No | No | Yes | Yes | No | Yes |
| MacDougall (2021) | No | Yes | Yes | No | No | No | Yes | Yes | Yes | No | No | Yes | Yes | Yes | No | Yes |
| Maddock (2023) | No | Yes | No | No | Yes | Yes | Yes | No | Yes | No | Yes | Yes | Yes | Yes | No | Yes |
| Magidson (2015) | No | No | Yes | Yes | Yes | No | Yes | No | No | No | No | Yes | Yes | Yes | No | Yes |
| Matsea (2022) | No | Yes | Yes | No | No | No | Yes | Yes | No | No | No | Yes | Yes | Yes | No | Yes |
| Maulik (2016) | No | No | Yes | No | No | No | No | No | No | No | No | No | No | No | No | No |
| Memiah (2022) | Yes | Yes | No | No | No | No | Yes | Yes | Yes | No | No | Yes | Yes | Yes | No | Yes |
| Mukherjee (2024) | No | Yes | No | No | Yes | Yes | Yes | Yes | Yes | No | Yes | Yes | Yes | Yes | No | Yes |
| Murphy (2024) | No | No | Yes | No | Yes | Yes | No | No | Yes | Yes | Yes | Yes | Yes | Yes | No | Yes |
| Murray (2013) | No | Yes | Yes | No | No | No | Yes | Yes | Yes | No | No | Yes | Yes | Yes | No | Yes |
| Mutahi (2024) | No | Yes | Yes | Yes | Yes | No | Yes | Yes | Yes | No | No | Yes | Yes | Yes | No | Yes |
| Mutiso, Musyimi (2018) | No | No | Yes | No | Yes | Yes | Yes | Yes | Yes | No | No | Yes | Yes | Yes | No | Yes |
| Mutiso, Gitonga (2018) | No | No | Yes | No | Yes | Yes | Yes | Yes | Yes | No | No | Yes | Yes | Yes | No | Yes |
| Naslund (2021) | No | Yes | Yes | No | No | Yes | Yes | Yes | No | No | No | No | Yes | Yes | No | No |
| Nguyen (2023) | Yes | Yes | Yes | No | No | Yes | Yes | Yes | Yes | No | No | Yes | Yes | No | No | Yes |
| O'Donnell (2022) | No | Yes | Yes | No | No | No | Yes | No | No | No | No | Yes | No | No | No | Yes |
| Orengo-Aguayo (2020) | No | No | Yes | No | No | No | Yes | No | Yes | No | No | Yes | Yes | Yes | No | Yes |
| Passchier (2019) | No | No | Yes | No | No | No | Yes | No | No | No | No | Yes | Yes | No | No | Yes |
| Perera (2020) | No | No | Yes | No | No | No | Yes | No | Yes | No | No | Yes | Yes | Yes | No | Yes |
| Petersen (2022) | Yes | Yes | Yes | No | Yes | No | Yes | Yes | No | No | Yes | Yes | Yes | No | No | Yes |
| Premji (2021) | No | No | Yes | No | No | No | Yes | Yes | No | No | No | Yes | No | No | No | Yes |
| Rai (2023) | Yes | No | No | Yes | No | No | No | Yes | No | No | No | Yes | No | No | No | Yes |
| Rivera (2008) | No | No | Yes | No | No | Yes | Yes | Yes | No | No | No | Yes | Yes | Yes | No | Yes |
| Sangraula (2021) | No | No | Yes | No | No | No | Yes | No | Yes | No | No | Yes | Yes | Yes | No | Yes |
| Sapag (2016) | No | No | Yes | No | No | No | Yes | No | Yes | No | No | Yes | Yes | No | No | Yes |
| Shidhaye (2019) | No | No | Yes | No | No | Yes | Yes | Yes | No | No | No | Yes | Yes | No | No | Yes |
| Singh (2021) | No | No | Yes | No | No | Yes | Yes | Yes | No | No | No | Yes | Yes | Yes | No | Yes |
| Sit (2022) | No | No | No | No | Yes | No | No | Yes | No | No | No | No | Yes | No | No | No |
| Spagnolo (2020) | Yes | No | No | No | Yes | No | Yes | No | Yes | Yes | No | Yes | Yes | Yes | No | Yes |
| Ssebunnya (2021) | Yes | No | No | Yes | Yes | Yes | Yes | Yes | Yes | Yes | No | Yes | Yes | Yes | No | Yes |
| Tinago (2021) | No | No | No | No | Yes | No | Yes | Yes | No | No | No | Yes | No | No | No | Yes |
| Triplett (2023) | No | Yes | Yes | No | No | No | Yes | No | No | No | No | Yes | Yes | No | No | Yes |
| vanderBoor (2024) | Yes | No | Yes | Yes | Yes | Yes | Yes | Yes | No | No | No | Yes | Yes | No | No | Yes |
| Wasil (2020) | No | No | No | No | Yes | No | Yes | Yes | No | No | No | Yes | Yes | No | No | Yes |
| Wieling (2017) | Yes | No | No | Yes | Yes | No | No | Yes | No | No | No | Yes | No | No | No | Yes |
